# Clarifying the Association Between Food Insecurity and Chronic Disease: Why Methods Matter

**DOI:** 10.1007/s13668-026-00782-0

**Published:** 2026-07-27

**Authors:** Daniel J. Arenas, Sourik Beltrán, Paul Okoyeh, Horace M. DeLisser

**Affiliations:** 1 Perelman School of Medicine, University of Pennsylvania, Philadelphia, PA USA; 2 Division of General Medicine, Beth Israel Deaconess Medical Center, Boston, MA USA; 3 The Mongan Institute, Massachusetts General Hospital, Boston, MA USA; 4 Wake Forest University Baptist Medical Center, Winston-Salem, NC USA; 5 Jordan Medical Education Center, 6th Floor, Building 421 3400 Civic Center Blvd, Philadelphia, PA 19104-5162 USA

**Keywords:** Food insecurity, Chronic disease, Depression, Anxiety, Hypertension, Diabetes, Dyslipidemia, Self-reported diagnoses, Clinical implications, Measurement methods

## Abstract

**Purpose of Review:**

Food insecurity (FIS), limited or uncertain access to adequate, nutritious food, is frequently cited as associated with various chronic health conditions. With the goal of determining how these data might influence public policy and/or clinical practice, this narrative review synthesizes methodological insights from prior systematic reviews and more recent studies to critically examine the associations between FIS and the conditions of depression, anxiety, hypertension, diabetes, and dyslipidemia.

**Recent Findings:**

Numerous studies, including meta-analyses, have found that individuals screening positive for FIS are also significantly more likely to screen positive for depression or anxiety using validated tools for establishing these conditions. In contrast, the associations between FIS and cardiometabolic conditions such as hypertension, diabetes and dyslipidemia have been inconsistent, with rigorous meta-analyses failing to demonstrate strong associations of FIS with these conditions. These variations in findings reflect the use of self-reported disease diagnosis in contrast to measurement of blood pressure or disease biomarkers to indicate disease presence/activity; regional/geographic differences in the populations studied; and potential confounding by other conditions.

**Summary:**

While there is consistent evidence for associations of depression and anxiety with FIS, the associations between FIS and cardiometabolic diseases are complex and much less clear. In furthering our understanding of these associations and their implications for policy and clinical care, future studies should (i) emphasize measurable indicators of disease presence/activity over self-reports of disease; (ii) give greater attention to regional and population heterogeneity to better contextualize the data; (iii) more consistently account for key covariates in cross-sectional and longitudinal studies; and (iv) assess respondents’ overall health-related worry or somatic symptom burden, when self-reports are used as indicators of disease.

**Supplementary Information:**

The online version contains supplementary material available at 10.1007/s13668-026-00782-0.

## Introduction

Food insecurity (FIS) refers to limited or uncertain access to adequate and nutritious food [1]. In the United States FIS is a concern, with approximately 13% of households experiencing FIS in 2022 [2]. In response, there have been significant efforts to identify effective interventions to mitigate FIS [3–9], largely due to the potential broad impact of improving population and individual health. Examples of these types of interventions include income supplementation, housing assistance programs, food retailer subsidies, medically tailored meals/groceries, produce prescriptions and farmers’ market vouchers [10].

It has been proposed that FIS may contribute to the onset or progression of mental health conditions such as depression and anxiety as well as the cardiometabolic diseases of hypertension, type 2 diabetes, and dyslipidemia, which remain leading causes of morbidity and mortality worldwide [11–14]. Proposed mechanisms for these FIS-induced clinical outcomes include social marginalization leading to chronic stress and associated neuro-hormonal changes in physiology, limited access to high quality food resulting in deficiencies in macro- and micronutrients and/or consumption of sodium-rich, caloric-dense, high-glycemic, processed foods, and a social experience that leads to less preventative, high quality medical care [10, 15–21]. These plausible mechanisms have been the impetus for substantial research and public health and policy efforts to improve food access and security.

Beyond public health and policy initiatives, the association between FIS and chronic disease also has potentially important implications for clinicians. Because large-scale randomized controlled trials and longitudinal studies on FIS and chronic disease are difficult to implement, cross-sectional studies remain the most numerous and salient sources of evidence. Thus, when clinicians seek to understand the relationship between FIS and chronic conditions, they are often limited to findings derived from these observational data. Even in the absence of established causality, such associations can still hold clinical significance, particularly when they reflect consistent relationships through shared social or biological pathways, such as the association of low HDL levels with adverse cardiovascular outcomes [22]. Thus, it is crucial that such associations, even before causality is proven, are measured rigorously and interpreted with careful attention to their generalizability.

While the literature frequently describes FIS as a cause of chronic disease, this relationship is likely more nuanced. Demonstrating that FIS is associated with an increased prevalence or incidence of a chronic disease is a challenging quantitative task for three reasons: (i) the contextual heterogeneity of FIS; (ii) numerous covariates; and (iii) variations in how chronic disease is defined or assessed. In elaborating on this further, it is important to first note that FIS can manifest in distinct ways, ranging from insufficient caloric intake to poor dietary quality, all of which is influenced by the geographic, cultural, and socioeconomic context [23–25]. This heterogeneity may result in competing mechanisms. For example, limited access to healthy foods may lead to greater consumption of high-calorie, high-glycemic, processed foods. In contrast, within some regions, settings, or among certain individuals, food insecurity may result in overall caloric restriction [23–27]. This raises the possibility that, in some contexts, such as different populations experiencing heterogeneous characteristics of FIS, differences in diabetes outcomes between food-secure and food-insecure individuals may not be readily detected [28].

Second, covariates make any analysis complex, as the dietary and metabolic effects of FIS rarely occur in isolation. FIS is frequently associated with elevated rates of depression, anxiety, and chronic stress [29, 30], which may influence chronic disease risk through both behavioral and physiologic pathways. Additional comorbid factors, such as a sedentary lifestyle [31, 32], further complicate the picture. Beyond the concern that differences in covariate distributions (e.g. higher stress in the FIS group compared to the food-secure group) can bias results, these factors may also modulate the observed effect size itself, amplifying or attenuating associations in ways that may be difficult to disentangle.

Finally, there is the issue of how the presence or activity of specific chronic condition is defined or measured, which is a major focus of this review. A large portion of the studies of the associations of FIS with chronic cardiometabolic conditions have employed self-reported diagnoses or medication use as surrogates for disease presence/activity, often necessitated by the large size of the study or limited resources. However, as the field has advanced, more studies have incorporated direct clinical measurements. When recent meta-analyses explicitly separated studies based on measurement methodology, studies relying on self-reported outcomes tended to show stronger associations between FIS and chronic disease risk, while those using measured indices such as blood pressure, glycated hemoglobin (A1c), or lipid panels often showed null or attenuated associations [28, 33, 34]. Despite these data, many original studies and reviews subsequent to the publication of those meta-analyses continue to fail to distinguish between self-reported and measured outcomes when interpreting or synthesizing findings. This is particularly concerning, as self-reported diagnoses may be vulnerable to recall bias when participants are asked to report chronic conditions without reference to recent clinical confirmation.

This narrative review synthesizes methodological insights from both prior systematic reviews and more recent studies examining the associations between FIS and several chronic conditions, including depression, anxiety, hypertension, type 2 diabetes, and dyslipidemia. Its central aim is to highlight how differences in the means by which disease presence is defined/established - self-reported versus biomarker-assessed - influence the strength and interpretation of these observed associations. Comprehensive meta-analyses of data through published through 2019 [28, 30, 33, 34] +-provide bases for critical analyses of subsequent studies to enable a review of emerging trends (See Section 1 of Supplementary Document for search strategies). Organized into condition-specific sections, this review illustrates how measurement methods shape not only academic interpretation but also the potential clinical relevance of these findings. By integrating these examples across conditions and time, the review aims to support the development of clearer, more interpretable evidence to inform both public health research and clinical care.

### FIS and Mental Health

We begin our discussion of chronic health conditions with depression and anxiety, which serve as useful “positive controls” in the literature on FIS and health. Among the conditions commonly studied in this field, the association between FIS and screening for mental health outcomes is one of the most consistently documented. Numerous studies, including meta-analyses, have found that individuals screening positive for FIS are significantly more likely to also screen positive for depression or anxiety [29, 30]. Several mechanistic processes are likely to contribute to the association of FIS with poor mental health [17, 18]. These include the psychological distress triggered by uncertainty around obtaining adequate and nutritious food, the social stigma associated with FIS, and internalized shame, hopelessness and social isolation. Further, nutritional deficiencies related to inadequate intake of essential nutrients including proteins, polyunsaturated fatty acids and key micronutrients (B-complex vitamins, iron, zinc, copper and magnesium) can adversely impact cognition, mood and energy levels.

Unlike other conditions discussed in this review, depression and anxiety are assessed without biomarkers. However, many studies go beyond binary self-reports of prior diagnoses and instead utilize validated mental health screening tools, such as the Patient Health Questionnaire (PHQ-9), Generalized Anxiety Disorder scale (GAD-7), and the Center for Epidemiologic Studies Depression Scale (CES-D). These tools, while based on self-report, are validated, structured tools, and are widely used in both clinical and research settings [35–38]. This distinction is important as these instruments offer a high level of methodological rigor compared to a single-question asking patients to recall prior diagnoses.

In 2019 the authors of this review published a meta-analysis of U.S. studies investigating the associations of depression and anxiety with FIS that involved screening nearly 1600 manuscripts [30]. In this report FIS was strongly associated with a positive depression screening result an association that was maintained across diverse populations, study designs, and screening tools [30]. Unlike other outcomes where measurement methods appear to influence the strength of association, the relationship between food insecurity and positive depression screens remained consistent regardless of the instrument used. While it is important to acknowledge that a screening result is not equivalent to a formal clinical diagnosis, these findings nonetheless suggest a higher mental health burden experienced by individuals who are food insecure.

Far fewer studies compared to that for depression have examined the association between FIS and anxiety as of our 2019 systematic review and meta-analysis [30]. Nevertheless, the available evidence still showed a strong association between FIS and screening positive for anxiety [30]. The distinction between screening and formal diagnosis must be emphasized, as clinical anxiety diagnoses encompass significant granularity and specificity—such as generalized anxiety disorder, illness anxiety disorder, panic disorder, and anxiety related to specific phobias—which may not be differentiated by general screening tools.

The strength and consistency of the association between FIS and screening for depression has clinical significance. While it remains difficult to establish a direct causal link, conditions that co-occur with high frequency may prompt additional screening if one of the conditions is identified [39–44]. In a similar way, the robust association between FIS and depression suggests that screening for depression may be appropriate in food-insecure individuals, comparable to the recommendations for depression screening in patients with type 2 diabetes [45].

An association between FIS and screening positive for anxiety and depression is also important for understanding the association between FIS and other chronic conditions. Previous studies have suggested that both anxiety and depression have an associations with hypertension and diabetes [46–50]. As such, the results of screening for these conditions could be considered potential covariates in studies examining the relationship between food insecurity and chronic disease outcomes. More broadly, the inclusion of survey items that measure an individual’s concern about their health such as those found in the SSD-12, may offer useful insights and clarify some of the discrepancies between self-reported and objective markers of disease presence/activity in the FIS literature.

Recent intervention studies have begun to explore whether addressing FIS can improve mental health outcomes. There is some evidence of improvements in PHQ-9 scores following efforts to reduce FIS [51, 52]. Although the available studies employ pre–post cohort designs, and could benefit from pre-registration, their results are promising. These intervention studies underscore a growing need for randomized controlled trials to determine whether reducing FIS can directly improve depression and related mental health outcomes. Further urgency is given by the evolving literature in the association between FIS and increased suicidal ideation [53–55].

### FIS and Hypertension

The association between FIS and elevated blood pressure is physiologically plausible and has drawn increasing attention in recent years. Several pathways—including diets high in sodium and low in nutrient quality, chronic psychosocial stress, and limited access to preventive care—suggest mechanisms by which FIS could contribute to hypertension [16, 21]. Beginning in 2020, the authors of this paper published a series of broad systematic reviews which screened over 1,300 abstracts with multiple reviewers to examine the relationship between FIS and several cardiovascular outcomes, including hypertension [33], type 2 diabetes [28] and dyslipidemia [34]. Importantly, the inclusion criteria were designed to capture a wide range of study types, including those using both self-reported hypertension diagnoses and objectively measured markers of disease.

Our initial study focused on hypertension [33], where we found an association of FIS with hypertension depended on the means employed to establish/define the presence of hypertension. Studies using self-reported hypertension consistently showed a significant positive association with FIS, while those employing blood pressure measurements—whether systolic, diastolic, or both—showed weaker effects and collectively did not demonstrate a significant association. This pattern held across multiple analytic approaches, including studies that dichotomized hypertension using clinical cutoffs and those that treated blood pressure as a continuous variable.

Although our systematic review in 2020 [33] distinguished between self-reported hypertension and objective measurements of blood pressure, several publications have cited that paper as evidence of an association between FIS and hypertension broadly (Table S1, Supplementary Information). For example, a 2022 narrative review referenced our findings, while only generally noting a link between FIS and cardiovascular risk [56]. Similar references appear in pediatric and public health reviews [7, 57], and primary data papers [32, 58–66]. In these citations, the means by which hypertension was defined in the meta-analyses were not addressed. These instances may reflect a broader challenge in the literature. As the field grows, the subtle differences between the approaches used to establish disease presence/activity can be difficult to capture in secondary citations. Fortunately, other studies have accurately interpreted the results of our meta-analysis and have explicitly highlighted the distinction between self-reported and objectively measured disease markers [67–76].

Although a systematic review of post-2019 literature is beyond the scope of this narrative review, a few noteworthy trends and recent data deserve mention (Table S2, Supplementary Information). Many primary studies published since 2019 have continued to rely on a self-reported diagnosis of hypertension [77–82]. Some studies report a positive association between FIS and self-reported hypertension [77, 80, 82], although the reliance on a self-reported diagnoses is not explicitly specified, while others find no significant association [78, 79, 81]. Among studies that objectively measure blood pressure (Table S3, Supplementary Information), results are also mixed, with some showing an association [73, 83, 84] and others do not [71, 76, 85–87]. In some cases, associations are observed for systolic but not diastolic pressure, or vice versa [88–90]. In one large study in Nepal, FIS was associated with a lower risk of hypertension [91]. These variabilities in results reinforces the need for studies that clearly specify measurement methods and stratify analyses to identify potential effect modifiers.

### FIS and Type 2 Diabetes

The mechanisms by which FIS might increase the risk of type 2 diabetes are nuanced and context-dependent, with potentially opposing processes simultaneously active. So while FIS is often accompanied by a lower caloric intake, potentially supporting glycemic control [92–94], these putative metabolic benefits may be countered by the fact that food insecure individuals are prone to rely on highly processed, calorie-dense, high-glycemic foods, which are typically more accessible, but may contribute directly to insulin resistance and type 2 diabetes [95, 96]. Further, chronic stress induced by FIS, with its associated neuro-hormonal changes in physiology, may be associated with poorer glycemic control [48].

Our systematic review by Beltran et al. of the association between FIS and diabetes, covering published literature up to 2019 [28], revealed patterns similar to those observed for hypertension. Specifically, peer-reviewed studies relying on self-reported diabetes diagnoses consistently demonstrated significant associations between food insecurity and diabetes. In contrast, studies employing objective biomarkers - such as fasting glucose or A1c levels often reported weak, inconsistent, or non-significant findings [28].

Unfortunately, the subsequent literature since the publication of Beltran et al., has not consistently maintained the critical distinction between self-reported and objectively measured hyperglycemia or diabetes in relation to FIS. Since 2021, several peer-reviewed manuscripts have cited Beltran et al. as evidence of a general association between FIS and diabetes without specifying the type of diabetes measurement used, thus potentially overstating the strength of the evidence (Table S4, Supplementary Information) [97–99]. That said, a number of recent studies have clearly emphasized the difference between self-reported diagnosis and objective measures of diabetes [69, 70, 100–102].

With respect to new data employing objective measures, NHANES-based analyses have shown significant associations between FIS and diabetes biomarkers [88] and Walker et al. noted in a subgroup of patients who did not self-report a previous diabetes diagnosis, FIS was significantly associated with elevated A1c [103]. Table S5 of the Supplementary Information provides further details on other studies, some in the general population [84] and others within specific groups such as women [83, 85, 86], mothers [86], diabetics [104], and patients with HIV [87]. The studies were from different countries such as Iran [83, 85, 89], Mexico [87], Turkey [84], and United States [90, 104]. Although these studies have yielded mixed results in the association between FIS and type 2 diabetes, and are heterogeneous in sample size, design, and findings, they do contribute valuable geographic and cultural context to the literature. Lastly, Table S6 in the Supplementary Information presents examples of primary data studies that used self-reported diabetes diagnoses. These studies suggest that, even when relying on self-reported measures, the association between FIS and diabetes may vary across populations and contexts.

### FIS and Dyslipidemia

It has been proposed that the dietary mechanisms linking FIS to hypertension and diabetes might also lead to unfavorable lipid profiles [105]. However, despite plausible mechanisms for an association of FIS with dyslipidemias, the current literature does not support such an association.

In our 2022 systematic review (which included studies published through 2019) [34], we found that the FIS-dyslipidemia literature was notably smaller than the bodies of evidence for hypertension or diabetes. From a research standpoint, dyslipidemia offers additional granularity. It includes several biomarkers, including triglycerides, HDL, LDL, and total cholesterol, each with their own clinical implications. The requirement and cost for laboratory testing may explain the smaller number of studies in this area, but from both a clinical and public health viewpoint, the possible association between FIS and dyslipidemia certainly warrants a deeper exploration.

Our meta-analysis in 2022 by Arenas et al. [34], found that, across all four lipid markers, there was insufficient evidence to support a strong association between FIS and dyslipidemia. This was in part due to the small number of available studies. A more recent systematic review by other authors, covering literature through October 2021, reached similar conclusions when looking at lipid biomarkers [106].

A brief overview of studies published since 2020 highlights some interesting findings. Table S7 of the Supplementary Information lists the studies, specific populations, and which lipid panels were measured. A large NHANES-based study by Sun et al. found significant associations between FIS and both lower HDL and higher triglycerides, although effect sizes were modest [88]. That study found no significant differences for LDL or total cholesterol. Other recent, but smaller, studies have shown associations between FIS and all lipid markers [85, 89], while others have reported mixed results, with associations for some markers but not others [83]. Still, other studies have found no associations across any of the lipid biomarkers [84, 86, 87, 90]. Even among studies using self-reported dyslipidemia, findings are inconsistent [81, 82]. Taken together, these studies indicate that a strong association between FIS and dyslipidemias has not been established.

## Conclusions

FIS is a significant public health concern both socially and clinically. In presenting this review, our goal is not to discount the broad, deleterious impacts of FIS, but rather to sharpen the tools we use to understand its effects such that future research can produce reliable and actionable results leading to effective public policy and clinical efforts that improve patient outcomes. We therefore propose that future efforts investigating the link between FIS and various mental health conditions and cardiometabolic diseases should focus on: (i) clearer characterization of FIS–chronic disease associations to avoid misleading clinical inferences; (ii) greater attention to population, regional, and cultural heterogeneity to better capture contextual variation; (iii) more consistent consideration and reporting of key covariates that are readily accessible in both cross-sectional and longitudinal studies; and (iv) assessing respondents’ overall health-related worry or somatic symptom burden, when self-reports are used as indicators of disease.

First, all the chronic conditions discussed in this narrative review - depression, anxiety, hypertension, type 2 diabetes, and dyslipidemia - are important from both clinical and public health perspectives. Observed associations between FIS and these conditions have the potential to inform decisions around screening, monitoring, and treatment intensity. However, except for depression and anxiety, the evidence base linking FIS to certain cardiometabolic conditions remains inconsistent - particularly when disease presence/activity are measured objectively. In this context, an overreliance on studies using self-reported diagnoses - without clear reference to or confirmation by physiological or laboratory assessments - risks overstating the strength and thus the clinical significance of these associations.

Second, overly generalized or imprecise findings may obscure the specific contexts in which the health impacts of FIS are most pronounced, potentially hindering the development of targeted interventions and regionally relevant research. Even among the post-2019 studies reviewed here that rely on self-reported diagnoses, associations between FIS and conditions such as hypertension, type 2 diabetes, and dyslipidemia vary across populations. This inconsistency underscores the need for caution when making broad claims about FIS-related health risks. Future research should more clearly differentiate between self-reported and objective assessments of disease and explore how associations differ by population subgroup and geographic setting. To diversify the evidence base, additional studies, both within the U.S. and globally, are needed. In particular, work done in the U.S. using alternative data sources such as electronic health records, longitudinal cohorts, or regional public health datasets could reduce redundancy and provide complementary, context-specific insights.

Third, as future studies evolve, it may not be possible for all analyses to account for every potential confounder. Nonetheless, methodological rigor involves not only the use of objective biomarkers, but also careful consideration of relevant covariates that may influence observed associations. Some covariates such as genetic predispositions, regional environmental exposures, and long-term lifestyle patterns are undoubtedly important, but they are often difficult to measure reliably in large, survey-based datasets. Others, however, represent more accessible opportunities for improving analytical clarity. Psychological factors such as depression and anxiety, which are independently associated with both FIS and chronic disease, are increasingly included in national surveys and can offer valuable insights when incorporated into statistical models. Similarly, behavioral variables like smoking status are commonly collected and may strengthen future analyses. Furthermore, individuals with more frequent healthcare encounters - often influenced by factors such as insurance status or comorbidities - may be more likely to receive a diagnosis regardless of true disease prevalence. This dynamic can introduce diagnostic opportunity bias, potentially inflating observed associations between FIS and a chronic disease. Consequently, future studies that explore healthcare utilization as a potential covariate could be extremely valuable.

Finally, in studies that rely on self-reported diagnoses, researchers may benefit from assessing respondents’ overall health-related worry or somatic symptom burden. Instruments such as the Somatic Symptom Disorder–B Criteria Scale (SSD-12) [107, 108] can offer valuable insights into how concerns about health might influence self-report behavior. These psychological factors may also relate to care-seeking patterns, potentially contributing to care-seeking or diagnostic opportunity bias. Incorporating such measures could help clarify discrepancies between self-reported and objectively measured outcomes.

A detailed consideration of how FIS was assessed in the studies that formed the basis of this review is beyond the scope of this paper. We would note, however, that the 2-item Hunger Vital Sign (HVS) has emerged as the most widely used validated FIS screen in the clinical setting. Importantly, the HVS is now available for use in electronic health records systems, typically in the context of a broader social determinants of health (SDOH) screening tool [109]. We believe that with the implementation of what we proposed above, these clinical trends will facilitate the integration into clinical practice of the data that emerges from the enhanced rigor of studies investigating the associations of FIS with mental health conditions and cardiometabolic diseases.

## Key References

- Sumsion RM, June HM, Cope MR. Measuring food insecurity: the problem with semantics. Foods. MDPI; 2023;12:1816.
  - ○ This paper provides an overview of current understandings, definitions and measures of food insecurity that can help to guide the work of academics, policy makers and other stakeholders in the field.
- Sharma A. Exploratory spatial analysis of food insecurity and diabetes: an application of multiscale geographically weighted regression. Annals of GIS. 2023;29:485–98. 10.1080/19475683.2023.2208199.
  - ○ This study investigated the association of food insecurity with diabetes within a local context by developing a socio-ecological regression analysis which modelled variation across both space and scale, focusing on four geographically connected states in the southern U.S. (Alabama, Arkansas, Mississippi and Tennessee). The author found that food insecurity was positively associated with diabetes, but the relation varied in magnitude and significance across space, with the strongest associations found in northwestern Arkansas, milder associations for central Mississippi and Tennessee and weaker associations for southern Alabama and eastern Tennessee.
- Owens C, Cook M, Goetz J, Marshburn L, Taylor K, Schmidt S, et al. Food is medicine intervention shows promise for engaging patients attending a safety-net hospital in the Southeast United States. Frontiers in Public Health. Frontiers Media SA; 2023;11:1251912.
  - ○ This paper describes the development and early outcomes of a multi-stakeholder, collaborative, “Food is Medicine” intervention aimed at addressing food insecurity for patients at risk for hypertension and diabetes.

## Supplementary Information

Below is the link to the electronic supplementary material.

Supplementary Material 1

## Data Availability

No datasets were generated or analyzed during the current study.
