# Supplementary material for: Clarifying the Association Between Food Insecurity and Chronic Disease: Why Methods Matter
Source: Curr Nutr Rep. 2026 Jul 27;15(1):64. doi: 10.1007/s13668-026-00782-0 (PMC13407713; doi:10.1007/s13668-026-00782-0)
Supplement: Supplementary file 1 — Supplementary Material 1 [file 13668_2026_782_MOESM1_ESM.pdf]

Clarifying the Association between Food Insecurity and Chronic Disease: Why Methods Matter
Supplementary Information

Daniel J. Arenas MD PhD[1], Sourik Beltrán MD[2,3],
Paul Okoyeh MD[4], Horace M. DeLisser MD[1]

[1]Perelman School of Medicine, University of Pennsylvania, Philadelphia, PA
[2]Division of General Medicine, Beth Israel Deaconess Medical Center, Boston, MA
[3]The Mongan Institute, Massachusetts General Hospital, Boston, MA
[4]Wake Forest University Baptist Medical Center, Winston-Salem, NC

Section 1: Search Strategy and Review Scope

This work is a narrative review, not a systematic one. Its primary objective is to synthesize and contextualize methodological variation—particularly the distinction between self-reported and objectively measured outcomes—in studies examining the relationship between food insecurity (FIS) and chronic disease.

Comprehensive systematic reviews and meta-analyses published through 2019 had already synthesized studies linking FIS to hypertension, diabetes, and dyslipidemia. These reviews employed broad search strategies across multiple databases and, importantly, stratified their findings by measurement method (e.g., self-reported diagnosis vs. biomarker-based definitions). Given the thoroughness and methodological clarity of those earlier efforts, we did not attempt to duplicate them. Instead, this narrative review builds upon that foundation by evaluating how methodological choices in post-2019 studies may shape interpretation, especially for clinical audiences.

To identify relevant recent literature, we conducted a targeted (but non-systematic) PubMed search in April 2025 using straightforward keyword combinations—"food insecurity" with "dyslipidemia," "hypertension," or "diabetes"—to capture some studies published through early 2025. We also identified additional studies through citation tracing and review of key references from prior systematic reviews and well-known papers in the field. Because this is a narrative review focused on methodological themes, our goal was not to be exhaustive but to illustrate how post-2019 studies reflect or diverge from earlier patterns, particularly in terms of outcome measurement.

Section 2: Supplementary Tables

This section highlights and organizes the post-2019 studies discussed in the main manuscript.

Year	Publication	Relevant text	Meta-analysis cited as
2022	Caceres et al.[1]	"Further, findings from systematic reviews food insecurity indicate that is associated with a higher risk for incident hypertension, type 2 diabetes, and CVD, as well as greater cardiovascular mortality [41–45]"	#41
2022	Kolli et al.[2]	"There is strong evidence that food insecurity is a risk factor for diabetes and hypertension, which are, in turn, risk factors for VI.[25,26,45,46]"	#46
2022	Robinson et al.[3]	" a meta-analysis by Beltrán et al.,[95] food insecurity was significantly associated with hypertension (odds ratio 1.44, 95% CI 1.16–1.79)."	#95
2023	Owens et al.[4]	"In the face of such barriers, numerous studies have documented associations between food insecurity and adverse health outcomes, including hypertension (9–11)"	#10
2023	Arzhang et al.[5]	"Furthermore, individuals experiencing food insecurity and poor-quality diets have a greater risk of diabetes and hypertension, which is a potential risk factor for vision impairment through increased inflammatory and oxidative stress products (10, 30, 31, 32, 33)."	#30
2023	Dillman et al.[6]	"Food insecurity impairs medication adherence because patients are confronted with a challenging situation in which they must either feed themselves or spend limited resources on antihypertensive medications.17-20"	#18
2023	Fong et al.[7]	"Past studies have also shown that the lack of regular access to enough safe and nutritious food is linked to a range of chronic health conditions, such as cardiovascular diseases [6,7], hypertension [2,8], diabetes [9,10], and physical frailty [11–13]."	#8
2023	Kota et al.[8]	"Previous studies suggest that food insecurity contributes to poor mental health conditions such as depression, stress and anxiety, suicidal ideation [8, 9], cardiovascular diseases [10], hypertension [11], and diabetes [12]."	#11
2023	Meyerovitz et al.[9]	" which is consistent with other studies' findings associating food insecurity with hypertension, cardiovascular disease, and poorer chronic disease management.9, 45, 46"	#45
2024	Onugha et al.[10]	"Similar to previous reports, our study observed a significant association between food insecurity and hypertension among U.S. adults,24,25 reinforcing the link between food insecurity and chronic diseases like hypertension.10"	#24
2024	Myers et al.[11]	"A large and growing literature has clearly established that food insecurity is strongly associated with adverse health outcomes, including poor overall health, cardiovascular disease, hyperlipidemia, stroke, kidney disease, non-alcoholic fatty liver disease, inflammation, chronic pain, pulmonary disease, and diabetes (Beltran et al., 2022)"	Beltran et al., 2022
2025	Barnes et al.[12]	"Hispanic households generally also have higher rates of food insecurity,23 which, in turn, is associated with having hypertension.24"	#24
2025	Chen et al.[13]	"Even worse, irregular dietary patterns disrupt metabolic health and increase the risk of obesity [17], malnutrition [18], hypertension [19], heart disease [2], and other chronic diseases [12,20],"	#19
2025	Hou et al.[14]	"Inadequate nutrition due to FI can contribute to inflammation, oxidative stress, and endothelial dysfunction, all of which are implicated in the development of hypertension[31]."	#31

Table S1. Examples of studies citing the systematic review by Beltran et al.[15] as evidence of an association between food insecurity (FIS) and hypertension, without noting that the original review found significant associations only for self-reported (not measured) hypertension.

Year	Publication	N	Population	Association with FIS	Relevant Information Found in
2021	Long et al.[16]	533	Native Hawaiian and Pacific Islander (NHPI)	Yes*	Table 3
2022	Sharedal et al.[17]	10,449	NHANES 2007-2016	No	Table 1
2022	Wu et al.[18]	157	Clients of a food pantry in Michigan	No	Text
2023	Dong et al.[19]	6,424	NHIS-2019-2020. Diabetic patients.	Yes	Table 1**
2024	Guedes et al.[20]	316	Seniors (>60 yrs old) in Brazil	No	Table 3
2025	Royer et al.[21]	3,676	NHANES2017-2018, 40 yrs and older	Yes	Table 3

Table S2. Examples of studies published since 2020 with data exploring the association between a positive food insecurity (FIS) screen and self-reported history of hypertension diagnosis. This table is part of a narrative review; studies were not identified through a formal systematic review process.

*: Association was significant only with very low food insecurity

**: Results shown for weighted sample sizes

Year	Publication	Measurement Method	N	Population	Association with FIS	Relevant Information Found in
2020	Hamedi et al.[22]	HTN defined as: SBP >= 140 or DBP >= 90 or use of anti-HTN drugs*	630	Women in Zabol, Iran	Yes	Table 3
2020	Sun et al.[23]	SBP and DBP from NHANES	27,188	NHANES 1999-2014	Systolic: No; Diastolic: Yes	Table 2
2021	Abdurahman et al.[24]	SBP and DBP	277	Public health centers in Tehran, Iran	No	Table 3
2022	Hashemzadeh et al.[25]	SBP and DBP	190	Women in Shiraz, Iran	Systolic: Yes; Diastolic: No	Table 3
2022	Joshi et al.[26]	HTN defined as: SBP >= 140 or DBP >= 90 or use of anti-HTN drugs	9,827	Nepal Demographic and Health Survey	Yes**	Table 3
2022	Sonnenblick et al.[27]	SBP and DBP from EHR, outpatient only. SBP >= 140 or DBP >= 90	180	Medicaid ACO patients; community health centers near Boston	No***	Table 2
2023	Caamaño et al.[28]	SBP and DBP	321	Mothers in Querentaro, Mexico	No	Table 3
2023	Dong et al.[29]	SBP and DBP	142	American-Indian/Alaska-Native youth in urban California	Systolic: Yes; Diastolic: No	Table 3
2023	Ing et al.[30]	BP measured by the participants	124	Parent/Guardian of a child enrolled in CHL. Hawaii	Yes	Table 2
2024	Bozdemir et al.[31]	HTN defined as: SBP >= 140 or DBP >= 90	175	Adults in Turkey	Yes	Table 2
2025	Christian et al.[32]	HTN defined as: SBP >= 140 or DBP >= 90	430	Women farmers in Ghana	No	Table 1
2025	Valerio et al.[33]	ICD10 codes and medications on EHR	479	HIV Clinical Cohort	No	Table 2

Table S3. Examples of studies published since 2020 with data exploring the association between a positive food insecurity (FIS) screen and objectively measured hypertension indicators. Unless otherwise noted, blood pressure measurements were conducted by the study investigators. This table is part of a narrative review; studies were not identified through a formal systematic review process.

CHL: Children's Healthy Living Center of Excellence

*: Definition of HTN as explained in the methodology section[22]

**: FIS was associated with lower risk of hypertension

***: Association dropped with covariate analysis

Year	Publication	Relevant text	Meta-analysis cited as
2023	Cai et al.[34]	" Food insecurity could alternatively increase the risks of adverse health conditions such as diabetes [37], hypertension [38]"	#38*
2024	Sohrabi et al.[35]	"Second, food insecurity is a significant cause of chronic stress, which potentially triggers cortisol release pathways and exacerbates disruptions in glucose tolerance and insulin sensitivity, influencing the development of diabetes [54]."	#54
2025	Kehm et al.[36]	"FI is associated with several established and emerging risk factors for HCC, including poor diet quality [37], diabetes [38,39,40],"	#40
2022	Salinas-Roca et al.[37]	"Inadequate nutritional habits or a deficient diet can lead to metabolic disorders and consequently the development of chronic non-communicable diseases (NCDs), such as cardiovascular disease (CVD) and diabetes mellitus (DM) [3,4,5,6,7]."	#7

Table S4. Examples of studies citing the systematic review by Beltran et al.[38] as evidence of an association between food insecurity (FIS) and diabetes (top 3 rows), without noting that the original review found significant associations only for self-reported—not objectively measured—outcomes. The bottom row shows one study citing the meta-analysis by Arenas et al.[39] on FIS and dyslipidemia, without distinguishing between self-reported and measured outcomes.

*: The diabetes meta-analysis is being cited for hypertension, perhaps the authors meant the citation for diabetes

Year	Publication	Method of Measurement	N	Population	Association with FIS	Relevant Information Found in
2020	Hamedi et al.[22]	Fasting blood glucose and post-prandial glucose	630	Women in Zabol, Iran	No	Table 3
2020	Sun et al.[23]	Fasting glucose from NHANES	27,188	NHANES 1999-2014	Yes	Table 2
2021	Abdudarahman et al.[24]	Fasting blood glucose	277	Public health centers in Tehran, Iran	Yes	Table 3
2021	Walker et al.[41]	A1c and self-report	35,216	NHANES 2003-2016	Yes	Table 1
2022	Hashemzadeh et al.[25]	Fasting blood glucose	190	Women in Shiraz, Iran	Yes	Table 2
2023	Caamaño et al.[28]	Fasting glucose and insulin resistance	321	Mothers in Querentaro, Mexico	Yes	Table 3
2023	Dong et al.[29]	A1c	142	American-Indian/Alaska-Native youth in urban California	No	Table 3
2024	Bozdemir et al.[31]	Fasting glucose, if high, step up to glucose tolerance test	175	Adults in Turkey	No	Table 2
2024	DeJesus et al.	A1c in EHR records	1,596	Diabetic patients in primary care clinics in Minnesota	No	Table 4
2025	Valerio et al.[33]	A1c and medications on EHR	479	HIV Clinical Cohort	No	Table 2

Table S5. Examples of studies published since 2020 with data exploring the association between a positive food insecurity (FIS) screen and objectively measured diabetes indicators. Unless otherwise noted, measurements were conducted by the study investigators. This table is part of a narrative review; studies were not identified through a formal systematic review process.

Year	Publication	N	Population	Association with FIS	Relevant Information Found in
2021	Long et al.[16]	506	Native Hawaiian and Pacific Islander (NHPI)	Yes*	Table 3
2022	Osborn et al.[40]	2,284	California Health Interview Survey (2012–2017)	Yes	Table 2
2022	Sharedal et al.[17]	10,449	NHANES 2007-2016	No	Table 1
2022	Wu et al.[18]	157	Asian Americans in Michigan	No	Text
2024	Guedes et al.[20]	316	Seniors (>60 yrs old) in Brazil	No	Table 3
2025	Royer et al.[21]	3,676	NHANES 2017-2018; 40 yrs and older	Yes	Table 3

Table S6. Examples of studies published since 2020 with data exploring the association between a positive food insecurity (FIS) screen and self-reported history of diabetes diagnosis. This table is part of a narrative review; studies were not identified through a formal systematic review process.

*: Association was significant only with very low food insecurity

Year	Publication	Method of Measurement	N	Population	Association with FIS	Relevant Information Found in
2020	Hamedi et al.[22]	TC, TG, HDL, LDL	630	Women in Zabol, Iran	Yes: TG; No: HDL, LDL, TC	Table 3
2020	Sun et al.[23]	TC, LDL, HDL, TG from NHANES*	27,188	NHANES	Yes: HDL, TG; No: LDL, TC	Table 2
2021	Abdurahman et al.[24]	TG, HDL	277	Public health centers in Tehran, Iran	Yes: TG; No: HDL	Table 3
2022	Hashemzadeh et al.[25]	TC, HDL, LDL, TG	190	Women in Shiraz, Iran	Yes: All	Table 2
2023	Caamaño et al.[28]	TC, HDL, LDL, TG	321	Mothers in Querentaro, Mexico	No: All	Table 3
2023	Dong et al.[29]	TG, HDL, LDL	142	American-Indian/Alaska-Native youth in urban California	No: All	Table 3
2024	Bozdemir et al.[31]	TC, LDL, HDL. Dyslipidemia diagnosis by labs and physician	175	Adults in Turkey	No	Table 2
2025	Valerio et al.[33]	EHR: Dyslipidemia as either high TC levels or use of lipid-lowering medications	479	HIV Clinical Cohort	No	Table 2

Table S7. Examples of studies published since 2020 with data exploring the association between FIS and objectively measured dyslipidemia indicators. Unless otherwise noted, measurements were conducted by the study investigators. This table is part of a narrative review; studies were not identified through a formal systematic review process.

References

1. Caceres BA, Bynon M, Doan D, Makarem N, McClain AC, VanKim N. Diet, Food Insecurity, and CVD Risk in Sexual and Gender Minority Adults. *Curr Atheroscler Rep*. 2022;24(1):41-50. doi:10.1007/s11883-022-00991-2

2. Kolli A, Mozaffarian RS, Kenney EL. Food insecurity and vision impairment among adults age 50 and older in the United States. *American Journal of Ophthalmology*. 2022;236:69-78.

3. Robinson CH, Chanchlani R. High blood pressure in children and adolescents: current perspectives and strategies to improve future kidney and cardiovascular health. *Kidney international reports*. 2022;7(5):954.

4. Owens C, Cook M, Goetz J, et al. Food is medicine intervention shows promise for engaging patients attending a safety-net hospital in the Southeast United States. *Frontiers in Public Health*. 2023;11:1251912.

5. Arzhang P, Jamshidi S, Aghakhani A, et al. Association between food insecurity and vision impairment among older adults: a pooled analysis of data from six low-and middle-income countries. *The Journal of nutrition, health and aging*. 2023;27(4):257-263.

6. Dillman L, Eichner J, Humienny A, et al. The Impact of Supplemental Nutrition Assistance Program (SNAP) Enrollment on Health and Cost Outcomes. *NEJM Catalyst*. 2023;4(6). doi:10.1056/CAT.22.0366

7. Fong JH. Risk factors for food insecurity among older adults in india: Study based on LASI, 2017–2018. *Nutrients*. 2023;15(17):3794.

8. Kota K, Chomienne MH, Yaya S. Examining the disparities: A cross-sectional study of socio-economic factors and food insecurity in Togo. *Plos one*. 2023;18(11):e0294527.

9. Meyerovitz CV, Juraschek SP, Ayturk D, et al. Social Determinants, Blood Pressure Control, and Racial Inequities in Childbearing Age Women With Hypertension, 2001 to 2018. *JAHA*. 2023;12(5):e027169. doi:10.1161/JAHA.122.027169

10. Onugha EA, Banerjee A, Vimalajeewa HD, et al. Dietary Sodium and Potassium Patterns in Adults with Food Insecurity in the Context of Hypertension Risk. *medRxiv*. Published online 2024. Accessed June 14, 2025. https://pmc.ncbi.nlm.nih.gov/articles/PMC10863033/

11. Myers KP, Temple JL. Translational science approaches for food insecurity research. *Appetite*. 2024;200:107513.

12. Barnes L, Kunta A, Ham T, et al. Patient-Level Factors Associated with Antihypertensive Prescribing Patterns in a Free Clinic Setting. *Journal of Student-Run Clinics*. 2025;11(1). Accessed June 14, 2025. https://www.journalsrc.studentrunfreeclinics.org/index.php/jsrc/article/view/501

13. Chen S, Li Z, Zhang Y, Chen S, Li W. Food Insecurity, Physical Activity, and Sedentary Behavior in Middle to Older Adults. *Nutrients*. 2025;17(6):1011.

14. Hou HL, Sun GX. Associations between food insecurity with gestational diabetes mellitus and maternal outcomes mediated by dietary diversity: A cross-sectional study. *World Journal of Diabetes*. 2025;16(2):95463.

15. Beltrán S, Pharel M, Montgomery CT, Lopez-Hinojosa IJ, Arenas DJ, DeLisser HM. Food insecurity and hypertension: a systematic review and meta-analysis. *PloS one*. 2020;15(11):e0241628.

16. Long CR, Narcisse MR, Bailey MM, Rowland B, English E, McElfish PA. Food Insecurity and Chronic Diseases among Native Hawaiians and Pacific Islanders in the US: Results of a Population-based Survey. *Journal of Hunger & Environmental Nutrition*. 2022;17(1):53-68. doi:10.1080/19320248.2021.1873883

17. Sharedalal P, Shah N, Sreenivasan J, et al. Trends in 10-year predicted risk of cardiovascular disease associated with food insecurity, 2007–2016. *Frontiers in Cardiovascular Medicine*. 2022;9:851984.

18. Wu TY, Bessire R, Ford O, Rainville AJ, Man Chong C, Caboral-Stevens M. Food Insecurity and Diabetes: An Investigation of Underserved Asian Americans in Michigan. *Health Promotion Practice*. 2022;23(1_suppl):67S-75S. doi:10.1177/15248399221116088

19. Dong T, Harris K, Freedman D, et al. Food insecurity and atherosclerotic cardiovascular disease risk in adults with diabetes. *Nutrition*. 2023;106:111865.

20. Guedes VF, Pereira MLAS, Pereira DB dos S, Souza AL de, Teles BKA, Pereira MHQ. Association between food insecurity and chronic non-communicable diseases in older adults covered by the family health strategy: a cross-sectional study in the Brazilian Northeast. *Revista Brasileira de Geriatria e Gerontologia*. 2024;27:e240033.

21. Royer MF, Rosas LG, King AC. Food insecurity and cardiovascular disease risk factors among U.S. adults. *BMC Public Health*. 2025;25(1):817. doi:10.1186/s12889-025-22031-9

22. Hamedi-Shahraki S, Mir F, Amirkhizi F. Food Insecurity and Cardiovascular Risk Factors among Iranian Women. *Ecology of Food and Nutrition*. 2021;60(2):163-181. doi:10.1080/03670244.2020.1812596

23. Sun Y, Liu B, Rong S, et al. Food Insecurity Is Associated With Cardiovascular and All-Cause Mortality Among Adults in the United States. *JAHA*. 2020;9(19):e014629. doi:10.1161/JAHA.119.014629

24. Abdurahman A, Bule M, Fallahyekt M, et al. Association of diet quality and food insecurity with metabolic syndrome in obese adults. *International journal of preventive medicine*. 2021;12(1):138.

25. Hashemzadeh M, Teymouri M, Fararouei M, Akhlaghi M. The association of food insecurity and cardiometabolic risk factors was independent of body mass index in Iranian women. *J Health Popul Nutr*. 2022;41(1):41. doi:10.1186/s41043-022-00322-w

26. Joshi S, Thapa BB. Socioeconomic risk factors of hypertension and blood pressure among persons aged 15–49 in Nepal: a cross-sectional study. *BMJ open*. 2022;12(6):e057383.

27. Sonnenblick R, Reilly A, Roye K, et al. Social Determinants of Health and Hypertension Control in Adults with Medicaid. *J Prim Care Community Health*. 2022;13:21501319221142426. doi:10.1177/21501319221142426

28. Caamaño MC, García OP, Rosado JL. Food insecurity is associated with glycemic markers, and socioeconomic status and low-cost diets are associated with lipid metabolism in Mexican mothers. *Nutrition Research*. 2023;116:24-36.

29. Dong L, D'Amico EJ, Dickerson DL, et al. Food insecurity, sleep, and cardiometabolic risks in urban American Indian/Alaska Native youth. *Sleep health*. 2023;9(1):4-10.

30. Ing CT, Clemens B, Ahn HJ, et al. Food insecurity and blood pressure in a multiethnic population. *International Journal of Environmental Research and Public Health*. 2023;20(13):6242.

31. Bozdemir E, Yuksel A. Food Insecurity and Cardiometabolic Risk among Turkish Adults: A Cross-Sectional Study. *Jurnal Gizi dan Pangan*. 2024;19(2):69-78.

32. Christian AK, Owu RT, Kretchy IA. Household food insecurity, sociodemographic and lifestyle risk factors associated with high blood pressure among women in farming communities in Ghana. *BMC Women's Health*. 2025;25(1):181. doi:10.1186/s12905-025-03713-3

33. Valerio LA, Rzepka MC, Davy-Mendez T, et al. Food Insecurity Prevalence and Risk Factors among Persons with HIV in a Southeastern US Clinical Care Setting. *AIDS Behav*. 2025;29(1):45-54. doi:10.1007/s10461-024-04497-7

34. Cai J, Bidulescu A. The association between chronic conditions, COVID-19 infection, and food insecurity among the older US adults: findings from the 2020–2021 National Health Interview Survey. *BMC Public Health*. 2023;23(1):179. doi:10.1186/s12889-023-15061-8

35. Sohrabi M, Amirkalali B, Gholami A, et al. The association of food insecurity with non-alcoholic fatty liver disease (NAFLD) in a sample of Iranian adults: a path analysis of a cross-sectional survey. *BMC Res Notes*. 2024;17(1):272. doi:10.1186/s13104-024-06923-4

36. Kehm RD, Vilfranc CL, McDonald JA, Wu HC. County-Level Food Insecurity and Hepatocellular Carcinoma Risk: A Cross-Sectional Analysis. *International Journal of Environmental Research and Public Health*. 2025;22(1):120.

37. Salinas-Roca B, Rubio-Pique L, Carrillo-Alvarez E, Franco-Alcaine G. Impact of health and social factors on the cardiometabolic risk in people with food insecurity: a systematic review. *International Journal of Environmental Research and Public Health*. 2022;19(21):14447.

38. Beltrán S, Arenas DJ, Pharel M, Montgomery C, Lopez-Hinojosa I, DeLisser HM. Food insecurity, type 2 diabetes, and hyperglycaemia: A systematic review and meta-analysis. *Endocrino Diabet & Metabol*. 2022;5(1):e00315. doi:10.1002/edm2.315

39. Arenas DJ, Beltrán S, Pharel M, Lopez-Hinojosa I, Vilá-Arroyo G, DeLisser HM. A systematic review and meta-analysis of food insecurity and dyslipidemia. *The Journal of the American Board of Family Medicine*. 2022;35(4):656-667.

40. Osborn B, Albrecht SS, Fleischer NL, Ro A. Food insecurity, diabetes, and perceived diabetes self-management among Latinos in California: differences by nativity and duration of residence. *Preventive Medicine Reports*. 2022;28:101856.

41. Walker RJ, Garacci E, Ozieh M, Egede LE. Food insecurity and glycemic control in individuals with diagnosed and undiagnosed diabetes in the United States. *Primary care diabetes*. 2021;15(5):813-818.
